# Fidelity and moderating factors in complex interventions: a case study of a continuum of care program for frail elderly people in health and social care

**DOI:** 10.1186/1748-5908-7-23

**Published:** 2012-03-22

**Authors:** Henna Hasson, Staffan Blomberg, Anna Dunér

**Affiliations:** 1 Vårdal Institute, Swedish Institute for Health Sciences, Lund University, P.O. Box 187, SE-221 00 Lund, Sweden; 2 Karolinska Institutet, Department of Learning, Informatics, Management and Ethics, Medical Management Centre (MMC), Stockholm 171 77, Sweden; 3 School of Social Work, Lund University, P.O. Box 23, SE-221 00 Lund, Sweden; 4 Department of Social Work, University of Gothenburg, P.O. Box 720, SE-405 30 Göteborg, Sweden

**Keywords:** Adherence, Adaptation, Process evaluation, Complex intervention, Implementation, Care chain, Elderly

## Abstract

**Background:**

Prior studies measuring fidelity of complex interventions have mainly evaluated adherence, and not taken factors affecting adherence into consideration. A need for studies that clarify the concept of fidelity and the function of factors moderating fidelity has been emphasized. The aim of the study was to systematically evaluate implementation fidelity and possible factors influencing fidelity of a complex care continuum intervention for frail elderly people.

**Methods:**

The intervention was a systematization of the collaboration between a nurse with geriatric expertise situated at the emergency department, the hospital ward staff, and a multi-professional team with a case manager in the municipal care services for older people. Implementation was evaluated between September 2008 and May 2010 with observations of work practices, stakeholder interviews, and document analysis according to a modified version of The Conceptual Framework for Implementation Fidelity.

**Results:**

A total of 16 of the 18 intervention components were to a great extent delivered as planned, while some new components were added to the model. No changes in the frequency or duration of the 18 components were observed, but the dose of the added components varied over time. Changes in fidelity were caused in a complex, interrelated fashion by all the moderating factors in the framework, i.e., context, staff and participant responsiveness, facilitation, recruitment, and complexity.

**Discussion:**

The Conceptual Framework for Implementation Fidelity was empirically useful and included comprehensive measures of factors affecting fidelity. Future studies should focus on developing the framework with regard to how to investigate relationships between the moderating factors and fidelity over time.

**Trial registration:**

ClinicalTrials.gov, NCT01260493.

## Background

Intervention research has seldom systematically documented how different intervention components have been implemented in practice [1]. Analysis of the implementation process and its fidelity is important in order to understand what specific reasons caused an intervention to succeed or fail [2-6]. This is especially relevant for complex interventions that consist of several active ingredients [7,8]. Otherwise, there is a risk of evaluating effects of a program that have been described but not fully implemented [8].

Implementation fidelity is often defined as the degree to which a particular program follows an original program model, i.e., the model that was intended to be used by the program developers [9]. Fidelity can act as a potential mediator of the relationship between interventions and their intended outcomes [6]. Several prior studies have demonstrated that the implementation fidelity affects how well the program succeeds; programs with high fidelity have had better outcomes than programs with lower fidelity [10-16]. Some programs only had significant effects in the high-fidelity samples as compared to the entire intervention group [17,18]. However, an intervention cannot always be implemented fully according to the program model, because local conditions may require some program adaptation [6]. Some authors argue that local adaptations improve the fit of the intervention to local context, and successful interventions are dependent on adaptations [11]. Others argue that program implementation can be flexible as long as the essential elements of an intervention are implemented with high fidelity. According to the Replicating Effective Programs (REP) framework, the core elements of an intervention should be standardized, but the mechanism by which these are operationalized can be changed to allow flexibility in implementation [19]. Two basic forms of program adaptation involve modifications of program content and form of program delivery [20]. The content can be changed by omitting, modifying, or adding components. Changes concerning the delivery can deal with the manner or intensity with which the intervention components are delivered [20]. Fixsen et al. [5] suggested that selection of staff with correct competence and high motivation, adequate training, coaching and support, as well as continuous program evaluation, together with enabling financial, organizational, and human resources policies, were components related to implementation with high fidelity. Other authors have suggested that reasons for program changes included staff desire to increase a sense of ownership and create a better fit between a program and local needs [11], as well as a desire to improve program results [21]. Others have suggested that poor staff training and uncommitted staff are reasons for non-adherence in implementation [21].

The Conceptual Framework for Implementation Fidelity [6] suggested that fidelity is influenced by moderating factors in participant responsiveness to a program, complexity of an intervention, facilitation strategies, and quality of delivery. The framework has later been modified [22] by an additional two moderating factors--context and participant recruitment. Context refers to surrounding social systems, such as structures and cultures of organizations and groups, and historical and concurrent events [1]. Participant recruitment covers aspects such as reasons for nonparticipation among potential participants, subgroups that were less likely to participate, and consistency of recruitment procedures among potential participants [23,24]. Participant responsiveness refers to how well participants respond to, or are engaged by, an intervention. It involves judgments by participants about the outcomes and relevance of an intervention [6]. Responsiveness refers both to individuals receiving the intervention and individuals responsible for delivering it. Complex interventions and interventions that were vaguely described are assumed to be more difficult to implement with high fidelity than simple interventions [25]. Adequate facilitation strategies increase opportunities for higher and more standardized fidelity. Quality of delivery concerns 'the extent to which a provider approaches a theoretical ideal in terms of delivering program content' [9]. The authors [6] also suggested that there are complex relationships at work between the moderators, which may further affect the relationship between an intervention and the implementation fidelity. Fixsen et al. [5] also suggested that the implementation components are compensatory in nature. For example, less training may be supplemented with greater amounts of coaching or careful selection, and very well-designed staff performance evaluations may compensate for less training and little coaching. In summary, The Conceptual Framework for Implementation Fidelity suggested that different moderating factors might affect, positively or negatively, the implementation process and its fidelity. These factors interact with each other, and the effect of one factor on fidelity might be influenced by another moderating factor. Currently, there is little empirical research on factors that influence fidelity [6,9,26-28], as most prior studies on fidelity have focused solely on adherence [17]. A need for studies that make sense of the fidelity concept and clarifies the function of factors affecting fidelity and their relationship to one another has been emphasized [6].

This study concerns a care continuum intervention for frail elderly people living in their own homes. Community-dwelling frail older people often receive care from many providers and they are frequently admitted to hospitals [29]. The transition from hospital to home is a vulnerable period of discontinuity and potential adverse events [30,31]. Integrated care programs have been used to reduce fragmentation and to improve continuity and coordination of care. Such programs are often complex in nature, including several care providers and professions, which might challenge the evaluation of the programs [32]. Prior studies on integrated care programs for elderly people have found positive effects on older peoples' medication consumption, satisfaction with care, and activities of daily living and quality of life [33-35]. However, to our knowledge, none of the prior studies have conducted a thorough analysis of implementation fidelity. The present study was conducted as a randomized control trial [36]. The first results of the study showed that the older people receiving the continuum of care intervention perceived higher quality of care than those receiving regular care. Program effects on participants' healthcare utilization, functional ability, activities of daily living, health-related quality of life, and life satisfaction will be presented in upcoming articles. A prior analysis of the intervention implementation has highlighted hindering and facilitating factors during the initial implementation [32]. The present study is a first attempt to analyze the implementation fidelity of the intervention. It aims to systematically evaluate implementation fidelity and possible factors influencing fidelity of a complex care continuum intervention for frail elderly people. The modified version [22] of The Conceptual Framework for Implementation Fidelity [6] is used.

The study has the following objectives:

1. To empirically test The Conceptual Framework for Implementation Fidelity.

2. To evaluate the level of implementation fidelity of the intervention.

3. To evaluate how different moderating factors in the framework affect the implementation fidelity.

## Methods

### Study design

The intervention study was a randomized controlled study with a total of 161 elderly participants divided into intervention and control groups. The intervention took place in one middle-sized municipality in Sweden. Evaluations were made of the possible effects of the intervention on the participants' capability to perform activities, their health-related quality of life, their satisfaction with care, and emergency care consumption at 3, 6, and 12 months after the baseline measurement. The present study had a longitudinal design using multiple qualitative methods to investigate the implementation process and its fidelity during the study period.

### Description of the intervention

The intervention consisted of developing a continuum of care model for frail older persons. The ambition of the program was to include all essential care providers, i.e., municipal health and social care, a university hospital, and primary care (PC). The intervention was a systematization of collaboration between a nurse with geriatric expertise situated at the emergency department (ED), the hospital ward staff, and a multi-professional team for the care of the elderly with a case manager (CM) in the community. The multi-professional team included a nurse (the CM), a qualified social worker, an occupational therapist, and a physiotherapist. The intervention was based on prior research on integrated care programs for elderly people and diagnoses of the local needs [36]. It was developed by researchers at the Vårdal Institute, in close collaboration with the local practices [36]. Table 1 presents the logic model of the intervention.

**Table 1:** The logic model of the intervention

Core inputs	Immediate Impacts	Short-Term Impacts	Impacts	Outcomes
Geriatric assessment at emergency department,	Contact between emergency department and municipality case manager	Municipality care will have increased information regarding the needs of the older person,	Possibilities for earlier discovery of problems,	Maintained functional ability,
Case manager and multi-professional team at the municipality care,	Increased contact between case manager and hospital ward	Increased contact between emergency healthcare and municipality social care,	Earlier care and rehabilitation efforts and changes in care and rehabilitation plans,	Increased life satisfaction,
Care planning after hospital discharge at older person's home	Case manager has early contact with older person at hospital,	Older people have more knowledge of whom to contact when they need help,	Better uptake of older people's viewpoints	Reduced number of visits to the emergency department,
	Case manager has early contact with older peoples' families,	Increased participation opportunities for older people and their families in care planning		Reduced number of stays in hospital wards,
	Continuous contact between case manager and older people			Higher satisfaction with municipality care and rehabilitation

The intervention is briefly described here and can been seen in more detail in Table 2. The inclusion and randomization of participants and the intervention program started at the ED. For the intervention group, the nurse with geriatric expertise made an assessment of the elderly patients' needs of rehabilitation, nursing, and geriatric care. The assessment was transferred to the next care provider (ward nurses and municipality multi-professional team) to be used as a basis for further care planning. The municipality's CM contacted elderly persons at the hospital ward, the ward staff, and relatives of the elderly person if this was approved by the elderly person and if any relatives were available. Relatives were offered support and help if needed and desired. The multi-professional team made a care plan at the participants' home (instead of at the hospital) after their discharge from the hospital or after visiting the ED. The results of the geriatric assessment made at the ED were used as a basis for this assessment and care planning. The planning was also done in consultation with the participants. All care providers, such as home help services and home nursing care, were informed regarding the plan made. The CM followed up the care plan within a week and had telephone contact with participants at least once a month. The CM was available to the participants and their relatives for questions and consultation if needed. The control group received conventional care and outcome evaluations. Access to a case manager or multi-professional team was not available in the present organization. Care planning was conducted at the hospital and no information transfer was made from the hospital to the municipality for patients discharged from the ED directly to their homes. A more thorough presentation of the intervention and the conventional care has been presented previously [22,36].

**Table 2:** Implementation fidelity for each intervention component and moderating factors affecting fidelity

Intervention component	The intervention component	Extent to which these were conducted	Moderating factor affecting fidelity
1	At the ED, a nurse with geriatric expertise makes an assessment of the patients' needs of rehabilitation, nursing, and care.	Seldom (made at wards not at the ED)	Recruitment

2	The geriatric assessment is transferred to the hospital ward for participants who are admitted to a ward.	Seldom (since assessment was made at the wards)	Recruitment

3	The nurse with geriatric expertise informs the community team that the patient has visited the ED, and whether he/she was transferred to a ward or returned home.	Always	

4	The geriatric assessment is sent to the CM and the multi-professional team in the municipality.	Always	

*For participants who are admitted to the hospital ward:*			

5	CM visits participants in the ward.	Always	

6	CM contacts a patient responsible nurse at the ward to get information about the estimated time at the ward.	Always	

*For participants discharged from the ward:*			

7	A patient responsible nurse at the ward contacts the CM before discharge.	Always	

8	Discharge plan is done in collaboration between CM, a qualified social worker, the patient, a nurse and physician at the ward.	Always	

*Participants coming home from ED or from a ward:*			

9	CM contacts participants and offers care planning.	Always	

10	CM initiates support for patients' relatives if necessary.	Always, when a participant has a relative and allows the contact, which is 10% of the participants	Participant responsiveness

11	CM and the multi-professional team make a care plan at the elderly person's home a couple of days after the discharge.	Always at home, 10% of planning not all team members participating	Context: resources for employment

12	The care plan is based on the results in the geriatric assessment.	Always	

13	All planning is done in consultation with the patient.	Always	

14	The team informs other care providers regarding the plan made.	Always	

15	CM follows up the care plan within a week (telephone or home visit).	Always, via telephone	

16	CM has telephone contact with participants once a month except in cases where more frequent contact is needed.	Always, if the participant wants this. 5% wanted to take the contact by themselves.	Participant responsiveness

17	The participants are advised that CM is available for problem solving and assistance during office hours.	Always	

18	Patient's GP is informed by letter that the individual is participating in the project.	Always	

*ED *emergency department, *CM *case manager, *GP *general practitioner

### Data collection

**Figure 1:**
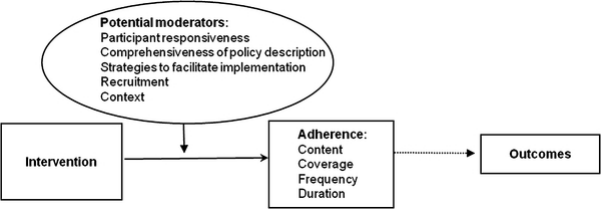
**Assessment of fidelity and moderating factors in the present study in accordance to the modified version of The Conceptual Framework for Implementation Fidelity (originally from Carroll et al.)**.

Data for the present study was collected from the start of the intervention (September 2008) until May 2010, which was the phase of the intervention when all of the participants had been included in the study. In accordance to the modified version [22] of The Conceptual Framework for Implementation Fidelity [6], data concerning the implementation fidelity and the moderating factors were collected. The measurement of implementation fidelity is a measurement of adherence, with its subcategories--content, frequency, duration, and coverage (dose). Thus, adherence relates to the content and dose of the intervention, i.e., whether the active ingredients of the intervention have been received by the participants as often and for as long as was planned [6]. Fidelity assessment should focus on all intervention activities if no analyses have been made of active ingredients of an intervention [5]. As such analyses had not been conducted in the present intervention, we evaluated adherence of all intervention components. The modified version of the framework identifies six moderating factors: participant responsiveness, comprehensiveness of policy description, strategies to facilitate implementation, quality of delivery, recruitment, and context (Figure 1). The framework suggested that all these factors should be evaluated systematically when conducting a process evaluation. However, it has been suggested [9] that quality of delivery can only be measured if an external benchmarking can be established. As the present study was the first evaluation of the care continuum model, no benchmarking was available. Thus, we evaluated the other five potential moderators.

Adherence and moderating factors were evaluated with observations, interviews, and document analysis. The second author of this paper conducted non-participant observations of the case managers' work practices. The CM was selected as an object of the observations because she had the most central role in the intervention. While observing her, the other actors, such as the multi-professional team, participating older people, hospital ward staff, geriatric nurses at the ED, and PC actors could also be observed. The observations were conducted once every six months at randomly selected three-day periods. A total of four observation visits were made during the study period. An observation protocol was developed based on the description of the planned intervention (Additional File 1). The observer reported how frequently (never, seldom, sometimes, often, always) the different intervention activities were conducted according to the plan. The observations were followed by questions to clarify the observed work practices and reasons for possible non-adherences. These clarifications were noted in a column for comments in the observations protocol.

Repeated interviews (once every six months, a total of four occasions) were conducted with key staff members at the operating level, i.e., nurses with geriatric expertise (two persons), case managers (two persons), the multi-professional team (four persons of which one is a municipality project leader), and a hospital project leader (one person). These individuals were the ones mainly involved in the delivery of the intervention. Interviews were also conducted with staff members who were less actively involved in the intervention. These persons were staff delivering support for family members (three persons, on two occasions) and representatives from hospital wards (four persons, at one occasion). Most of the interviews were conducted with two respondents from the same profession simultaneously. Thus, a total of 27 interviews were conducted with 16 actors at operating level. Workplace managers were interviewed but not included in the present analysis because the focus is on practical implementation of the intervention activities. The researchers contacted potential respondents with an e-mail that consisted of information about the project and the planned interview. It was emphasized that the participation was voluntary. Before the interview started, respondents were given more information about the study and the consent forms were signed. All interviews were conducted by one or two of the authors of the paper; they were semi-structured and lasted between 45 and 75 min. A general interview guide was developed focusing on: respondents' current work and role in the project including possible changes in these; experience of facilitation, feedback, and support; perceptions of organizational or other factors facilitating or hindering their work or the project in general; perceptions of the intervention content and the relevance/benefits for the participants; and expectations concerning the project and its effects (Additional File 2). This was used as a basic guide for all interviews. Additional questions were asked in some of the interviews in order to follow up earlier observations. The interviews were recorded and were later transcribed.

The researchers also gathered all project documentation from the participating organizations. The hospital and municipality project leaders were also asked to keep an ongoing work diary concerning the project activities, factors affecting the implementation of the intervention, and possible changes in the content or delivery of the intervention. A total of 119 documents were gathered; these included the project leaders' work diaries and notes (15 documents), minutes of meetings (101 documents), and project information letters for collaborators (three documents).

### Data analyses

The notes in the observation protocols were discussed by the first and second author after each observation visit. Delivery of each intervention component was discussed, and the level of adherence was determined. The interview and document data were analyzed independently by the two authors using content analysis [37]. The authors compared the interview and document data to the findings of the observed data of adherence. All non-adherences or obscurities in the data concerning the actual delivery of the intervention components were further investigated with specific interview questions in the next interview with the key stakeholders. The interview and document data was also analyzed to identify factors affecting fidelity. The Conceptual Framework for Implementation Fidelity was used as a coding scheme for the analysis. The interviews were categorized based on the moderating factors: participant responsiveness, comprehensiveness of policy description, strategies to facilitate implementation, recruitment, and context. The analysis started with a reading of each transcript independently by the first and second authors. The two authors coded the texts according the moderating factors and a comparison was made between the two codifications. For instance, the interview respondents described the intervention as highly relevant for the target group and believed it to have great impact on older peoples' health. This was coded as participant responsiveness. The few (less than 10%) differences in codifications that occurred were discussed among the authors. After the discussions, 100% agreement was obtained on the codifications of the moderating factors. At the end of the study period, all of the observation protocols were compared over time by the two authors. A general level of fidelity was determined for each component.

### Ethical approval

Ethical approval was granted by the Gothenburg University (Dossier number 413-08).

## Results

### Adherence

#### Content

A total of 16 of the 18 intervention components were always or most often delivered as these were described in the program protocol (Table 2). The two components not delivered according to the program plan concerned the geriatric assessment at ED. Recruitment and geriatric assessment was most often conducted in the wards instead of ED (component 1, Table 2). Consequently, the wards did not use the geriatric assessment as the basis for their care planning as it was transferred to them when patients were already in the wards (component 2, Table 2). The three intervention components most often delivered according to the plan concerned CM not having contact with all of the participants once a month (component 10, Table 2), not initiating support for all participants' relatives (component 17, Table 2) and not all team members always participating in care-planning meetings (component 11, Table 2).

Non-adherence also dealt with components that were added to the model. Team members also started, in addition to plan rehabilitation, to conduct the rehabilitation because the original rehabilitation staff had long waiting lists. The team also started to conduct six-month follow-up meetings with all participants, which was not planned in the original model. According to the municipality work praxis, every elderly person receiving home help services or home nursing care had a six-month follow-up. Thus, the team decided to conduct similar checkups even for those not having any services. CM had telephone contacts with relatives. She was very helpful and friendly, and it is possible that these telephone calls could have functioned as support for these relatives. In addition, the participants were allowed to contact the CM even after the 12-month period that originally was the intervention time for each participant.

### Frequency and duration (dose)

No changes in the frequency or duration of the 18 components were observed. However, dose of the added components varied over time. For instance, at middle stages of the project, the rehabilitation staff in the team received more resources, and they started to offer participants rehabilitation. At the end of the study, the CM and the team received a heavier workload as the total number of participants increased. The six-month follow-up evaluations were first conducted via telephone instead of home visit and after a while, no six-month follow-ups were offered to participants without any municipality elderly care services. To finish the official 12-month study period, the team first organized a home visit for the participants. However, during heavier workload periods this was conducted via telephone.

### Coverage

The project leaders' notes showed that a total of 340 persons who met the inclusion criteria were asked to participate. Of these, 159 (47%) individuals declined to participate.

### Moderating factors

#### Recruitment

All staff respondents and the project documentation indicated that participant recruitment was problematic. It was difficult to find individuals that fulfilled the inclusion criteria. The recruitment procedure took a long time to conduct, resulting in many elderly people not having sufficient energy for this procedure. There was seldom time at the ED for conducting the recruitment procedure and the geriatric assessment. This meant that the participants were recruited from the hospital wards instead of the ED. The geriatric assessment was also made in the wards instead of the ED. Patients leaving the ED without a hospitalization were not always possible to recruit to the study. The staff respondents experienced that the frailest people refused to participate, and assumed that the comprehensive procedure was the reason why these individuals did not have sufficient energy to get involved with the project. The project documentation also showed that the older individuals who declined to participate gave most often 'the project seems too demanding' (n = 76) and 'I'm too ill to participate' (n = 12) as reasons for not participating.

#### Participant responsiveness

The older persons' preferences and wishes were reasons for not always delivering the components concerning the CM's contact with the participants and their relatives. The CM was supposed to contact all participants at least once a month and contact participants' relatives to offer them support. A total of 5% of the participants wanted to contact the CM by themselves. Thus, the frequency of the contact was determined by the older participant and was not always once a month as was planned in the program description. Most of the older participants did not want the CM to contact their family members because they were concerned that this would burden the families. On the other hand, one fundamental component of the intervention was that all planning should be conducted in collaboration with the participant. Thus, according to this component, the intervention had high adherence.

High staff responsiveness seemed to be one of the main reasons for adding components to the intervention. All staff respondents expressed high enthusiasm about the project and about their own roles in it. Some of the respondents also had previous positive experiences of working with care continuum models for the elderly. The project staff was also proud of the project and voluntarily presented it at local and national conferences. The above-mentioned added components were conducted as staff members were highly engaged in their work; they believed that the intervention was relevant and that it had potential for reaching good outcomes for older people. The staff wished to further improve the benefits for the participants by adding components such as rehabilitation and follow-up meetings. On several occasions, the project staff received positive feedback from the participants and their families, which gave them further assurance that their work was valuable.

#### Context

Some contextual factors had a direct impact on fidelity. Financial resources for the employment of the rehabilitation staff in the team fluctuated during the project. This meant that during a period of fewer resources not all rehabilitation staff members could attend all care planning meetings (component 11, Table 2). Another impact of contextual factors concerned support for participants' relatives. The formal support available at the municipality focused on relatives of people with dementia and did not therefore suit the relatives in the project. Little formal support for relatives was available during most of the project time. Later on during the project, the municipality widened the target group, and some support for project relatives was available. Some contextual factors affected the adherence by adding intervention components. PC had a concurrent project where physicians and nurses carried out home visits to elderly individuals. The team in the present project established collaboration with that project and could offer those services to the participants during some months of the project.

Some of the contextual factors had more indirect impact on the implementation. The importance of having positive experiences from similar projects was an important driving force at the initial stage of the project. All staff respondents frequently referred to their prior experiences and used those to market the current project to collaborations. Prior work practices also affected co-workers' attitudes towards the project. Hospital physicians initially expressed concerns for patient safety because the care planning was conducted at patients' homes and not at the hospital ward as usual. Because these concerns were responded to with adequate information from the project team, no consequences for fidelity were observed. Other contextual factors affected the work of project staff, but did not have an impact on fidelity. Ongoing changes--such as a new IT program, remodeling at the ED, reorganization of the hospital organization, and new workplace leaders--created uncertainty among project staff and made it more difficult to accomplish their everyday work, but did not seem to affect fidelity.

### Complexity and facilitation strategies

Initially, all staff respondents expressed that they had received little information from the program designers concerning the project and the work descriptions, which were experienced as unclear. Facilitation at the initial stage was reported to be limited. Some of the respondents, especially at the hospital, thought that this was problematic, while others, mostly at the municipality site, seemed satisfied with the freedom to act according to their own judgment. These interviewees reported that they had taken more active roles in the project because no detailed information or facilitation was available. During the later phases of the study, the respondents experienced more information and facilitation. Some respondents also perceived the feedback from project steering groups and their work leaders as limited, which bothered some of the staff, while others stated that the limited feedback did not disturb them.

## Discussion

Comprehensive, longitudinal data material showed that the level of the fidelity of this complex intervention generally was high. A total of 16 of the 18 intervention components were always or most often delivered according to the original plan. However, some non-adherence was also observed, including components that were not delivered, were modified, and were added to the original. The different moderating factors in the Conceptual Framework for Implementation Fidelity all affected the fidelity in a complex, interrelated way. The effects of the moderating factors on fidelity also changed over time, which further illustrates the challenges of evaluating impact of factors influencing fidelity. The Conceptual Framework for Implementation Fidelity [6,22] was in general found to be empirically useful. The strengths, limitations, and the future use of the framework are discussed below.

Measurement of the four dimensions of adherence (content, frequency, duration, and coverage) included in the Conceptual Framework for Implementation Fidelity was found to be extensive and challenging, but also useful. First, some flexibility existed in the interpretation of the intervention components and delivery descriptions, which complicated the evaluation of adherence. Continuous discussions needed to be carried out in order to clarify each component. Standardization of core components and their delivery has also been emphasized by other authors [19]. It is challenging to describe content and delivery of several components so that no unclearness exists. Perhaps future studies could take into consideration the four adherence dimensions when formulating descriptions of intervention components and delivery. This could help to specify content, frequency, and duration for each component.

The last adherence dimension, coverage, was especially useful in the present study because almost half of the potential participants declined to participate. Many prior studies have not evaluated coverage [24], which makes it difficult to determine to what population the findings are generalizable. The moderating factor, recruitment, was found useful because it provided information on factors affecting coverage. Another challenge concerning the evaluation of adherence was the fact that no standards exist for what is the optimal degree of adherence. We considered high adherence only when the components were always or most often delivered as planned concerning content, frequency, and duration. There is also no agreement on whether and how to weight fidelity of the different intervention components, i.e., whether high fidelity for core component compensates for low fidelity for less important components. It is recommended that further studies discuss and define acceptable levels of adherence for the four adherence dimensions.

The findings also showed that non-adherence also dealt with components that were added to the model. Therefore, all measurements of adherence, such as fidelity protocols, should also include categories for additional components. Our analysis showed that staff did not reflect and recover components they had added, which could make it difficult to capture these in a protocol or interview. Therefore, it is strongly recommended that observations be used repeatedly to measure adherence and added components.

We found that staff enthusiasm for the project (responsiveness) was high, and this seemed to be a reason for adding components to the intervention. These additional components were in line with the theoretical ideas of the intervention, and no contradictory components were added. It seems that a desire to give the best possible care for the participants was a driving force for adding components. This is in line with Fraser et al. [21], suggesting that a desire to improve program results can be a reason for local intervention adaptations. Fixsen et al. [5] highlighted that understanding the principles of intervention core components may allow for flexibility in form without sacrificing the function associated with the components. We also found that some contextual factors in terms of merging services with concurrent projects and additional resources enabled the staff to add components to the present intervention. Thus, contextual factors enabled the additional components, but high staff responsiveness determined that the components were actually added. Staff with lower enthusiasm would perhaps not have added the components although contextual factors made that possible. Some authors have suggested that local additions to an original model tended to enhance effectiveness [11]. Effectiveness of additional components was not the focus of the present analysis, but we suggest that future studies should investigate the possible positive (and/or negative) impact of staff responsiveness and added components on program outcomes.

Contextual factors such as organizational routines were often reasons for not delivering or modifying components. For instance, the formal support for the relatives at the municipality was not targeting relatives of the present project, and therefore no formal support for relatives could be offered. In addition, staff enthusiasm about the project made them add components, but contextual factors such as increased workloads made them remove these in order to focus on the original components. This is a classical situation in organizational intervention research where interventions are not conducted in a vacuum. The longitudinal analysis revealed how the staff strived to strike a balance between resources and workloads on the one hand and staff willingness to deliver high quality care on the other hand. Fixsen et al. [5] suggested that high fidelity practices is best achieved when implementation is well-supported by strong organizational structures and cultures. This is most certainly valid, but difficult to achieve in practice when dealing with complex organizational interventions during a longer time period. In our case, the project had strong leadership support and the content of the intervention was developed in collaboration with the participating practices in order to develop a program that would suit the local context [36]. Participating organizations change leaders, reorganize their units, and get involved in new projects, and these actions make it difficult to plan in advance. This further emphasized the importance of longitudinal, systematic analysis of implementation fidelity in connection with an intervention study.

We also found that participant responsiveness,' i.e., elderly peoples' preferences, was a reason for not delivering components. The CM was supposed to contact all participants at least once a month to check their status. However, some participants wanted to contact the CM by themselves and as often as they wished. Prior studies have shown that intervention components that are not in line with recipients' wishes are most often not delivered [38].

Most of the respondents experienced the intervention as a complex program, the description of the intervention as vague, and the initial facilitation as limited. This is in line with prior studies reporting initial confusion in project work [39]. While some described the lack of clear descriptions in the initial intervention phase as frustrating and hindering, others experienced it as positive, because it gave them the possibility for individual interpretations of the intervention. Especially the municipality staff, which had long experience of working in similar projects, reported that they took a more active role and enjoyed the freedom to act according to their own judgment. It seems that the experiences of complexity and lack of initial facilitation did not impact fidelity, which is contradictory to prior studies suggesting that simple interventions and interventions with detailed descriptions are more likely to be implemented with high fidelity [6]. In this study, the staff was highly responsive to the intervention, which may have functioned as a driving force for them to solve complicated practical issues and take a more active role in the implementation. It is possible that unmotivated staff would not have made the same efforts if they had experienced limited facilitation. Some prior studies have reported staff to be more engaged, motivated, and effective when they feel they are exercising their judgment and expertise [40,41]. With this approach, the staff is not expected to follow process protocols exactly, but rather work according to their own judgments of what fits with the client characteristics and context and the program theory [42]. Implementation components, such as training, need to be standardized, but also flexibly adapted to different provider levels of experience [43]. In line with that approach, our findings suggest that individual and organizational differences in prior experiences and responsiveness to the intervention are important to consider by those delivering an intervention when developing work descriptions and planning facilitation activities.

Our findings emphasize the interrelationship that the moderating factors can have with each other and the fidelity. For instance, staff experiences of prior similar projects were to a great degree affecting their responsiveness to the present project, which in turn influenced preferred level of details in work descriptions and facilitation strategies. Many contextual factors also hindered and facilitated the work of the project staff, while the impact of these factors on fidelity seemed to be modified by the other moderating factors such as staff responsiveness. Fixsen et al. [5] suggested that the interactive implementation drivers compensate for one another so that a weakness in one component can be overcome by strengths in other components. Based on the results of the present study, it seems that staff willingness to deliver the program with high fidelity and participants' willingness to receive the components were the fundamental conditions for implementation of the program. Factors of particular importance for fidelity were staff and participants' responsiveness to the intervention on one hand, and the enabling and hindering contextual factors on the other hand. Implementation fidelity was shaped by the staff's commitment to the intervention program, as well as their ability to perform its content within the resources at hand. A staff with high responsiveness was also willing to overcome potential obstacles, such as contextual factors. These factors are recommended as first steps for evaluation of factors affecting fidelity. It is suggested that more research is needed for investigating the relationship between the moderating factors and fidelity.

The previously proposed [6,22] Conceptual Framework for Implementation Fidelity was a useful tool for organizing the data collection of adherence and moderating factors. It covered factors causing non-adherence, suggesting that these factors are comprehensive measures of factors affecting fidelity. However, the framework does not provide any guidance for how to investigate the interrelations between the moderating factors. It is suggested that the framework be further developed or used together with other models to examine the relative impact of the moderating factors on each other and fidelity longitudinally.

### Methodological discussion

The main strengths of the study were the use of three different data collection methods and the longitudinal design. In line with suggestions from other authors [44,45], the different data sources complemented each other and offered reliable results. The direct observations were especially valuable. A longitudinal analysis allows the researcher to track the development of the program over time, providing a more thorough understanding [44]. Some authors [45] have suggested that fidelity also needs to be measured in control groups. In the present study there was no possibility that the intervention components could have been delivered to controls due to organizational routines. The control group received care planning at the hospital and did not have any CM or a multi-professional team to contact in the municipality. Thus, after a careful evaluation, a decision was made that the research resources were not to be put into the evaluation of the control group. One limitation is also that elderly participants were not interviewed because their respondent burden was considered too high. Finally, the intervention was conducted in local practice, but in a research context. Thus, it is possible that the factors affecting fidelity in this project are not totally comparable to real-life situations, because support from researchers was offered. Nonetheless, as Dane and Schneider [10] point out, understanding fidelity under the research conditions is a first step to understanding program fidelity. The next step would be to study the implementation of the intervention after the research program.

## Conclusions

The Conceptual Framework for Implementation Fidelity was an empirically useful tool to collect and analyze data concerning the adherence. It also included comprehensive measures of factors affecting fidelity and provided guidance for analyzing the moderating factors. However, a complex interrelationship existed between the moderating factors, and the framework provided limited guidance for how to investigate the relations between the moderating factors over time. It is suggested that this framework be further developed or used together with other models to examine the relative impact of the moderating factors on each other and fidelity longitudinally.

## Competing interests

The authors declare that they have no competing interests.

## Authors' contributions

HH, SB, and AD designed the study and collected the data. HH and SB did the main analyses of the data with help from AD. HH drafted the manuscript with help from SB and AD. All authors read and approved the final manuscript.

## Supplementary Material

Additional file 1 **The observation guide**. The observation guide used in the study. Click here for file

Additional file 2 **The interview guide**. The general interview guide used in the study. Click here for file
