# Supplementary material for: Fidelity and moderating factors in complex interventions: a case study of a continuum of care program for frail elderly people in health and social care
Source: Implement Sci. 2012 Mar 22;7:23. doi: 10.1186/1748-5908-7-23 (PMC3342887; doi:10.1186/1748-5908-7-23)
Supplement: Additional file 1 — The observation guide. The observation guide used in the study. [file 1748-5908-7-23-S1.DOC]

**Additional file 1. The observation guide**

|  | **Extent to which these were delivered (always, often, sometimes, seldom, never)** | **Comments** |
| --- | --- | --- |
| At the ED, a nurse with geriatric expertise makes an assessment of the patients’ needs of rehabilitation, nursing, and care. |  |  |
| The geriatric assessment is transferred to the hospital ward for participants who are admitted to a ward. |  |  |
| The nurse with geriatric expertise informs the community team that the patient has visited the ED, and whether he/she was transferred to a ward or returned home. |  |  |
| The geriatric assessment is sent to the CM and the multi-professional team in the municipality. |  |  |
| *For participants who are admitted to the hospital ward:* |  |  |
| CM visits participants in the ward. |  |  |
| CM contacts a patient responsible nurse at the ward to get information about the estimated time at the ward. |  |  |
| *For participants discharged from the ward:* |  |  |
| A patient responsible nurse at the ward contacts the CM before discharge. |  |  |
| Discharge plan is done in collaboration between CM, a qualified social worker, the patient, a nurse and physician at the ward. |  |  |
| *Participants coming home from ED or from a ward:* |  |  |
| CM contacts participants and offers care planning. |  |  |
| CM initiates support for patients’ relatives if necessary. |  |  |
| CM and the multi-professional team make a care plan at the elderly person’s home a couple of days after the discharge. |  |  |
| The care plan is based on the results in the geriatric assessment. |  |  |
| All planning is done in consultation with the patient. |  |  |
| The team informs other professionals and care providers regarding the plan made. |  |  |
| CM follows up the care plan within a week, via telephone or home visit. |  |  |
| CM has telephone contact with participants once a month except in cases where more frequent contact is needed. |  |  |
| The participants are advised that CM is available for problem solving and assistance during office hours. |  |  |
| Patient’s GP is informed by letter that the individual is participating in the project. |  |  |
