# Supplementary material for: Fidelity and moderating factors in complex interventions: a case study of a continuum of care program for frail elderly people in health and social care
Source: Implement Sci. 2012 Mar 22;7:23. doi: 10.1186/1748-5908-7-23 (PMC3342887; doi:10.1186/1748-5908-7-23)
Supplement: Additional file 2 — The interview guide. The general interview guide used in the study. [file 1748-5908-7-23-S2.DOC]

**Additional file 2. The general interview guide**

**Background**

Could you tell about your background in terms of education and work experience?

Could you tell about your current work?

**Role and work in the Care Continuum project**

What is your role in the Care Continuum project? (in the follow-up interviews: Have there been any changes in your role in the Care Continuum project? What are the reasons for the changes?)

Could you describe the work you are currently doing in the project? (in the follow-ups: Have there been any changes in your work? What are the reasons for the changes?)

How does the work in the project differ from the normal care concerning the older people? (what are the major differences in the care of the older persons)

Do you have some work descriptions for your work? (if yes, these are collected to the document analysis) (in the follow-up interviews: Have there been any changes in your work descriptions?)

How do you document the work you are doing? (all documentation mentioned is collected to the document analysis)

Have there been any other changes in the Care Continuum model?

Why were these changes made?

**Information, facilitation**

How do you experience the information you have received concerning the Care Continuum project?

Do you feel sufficiently informed about what you are expected to do?

How do you experience the facilitation, support and feedback you have received concerning your work in the Care Continuum project?

**Factors facilitating or hindering the work or the project in general**

Could you describe any factors that hinder or facilitate your work in the project?

What conditions do you see as critical to the project’s success?
Are there some factors hindering or facilitating the implementation of the project?

Do you have any concerns regarding the implementation of the project?

**Relevance/benefits for the participants**

What are the main issues the project can contribute to the care of older people?

What activities in the project lead to the desired effects?

**Expectations concerning the project and its effects**

Do you have any expectations for the project as a whole?

What do you think are the main benefits of this project?

Is there anything else that you would like to elaborate on or share regarding the Care Continuum project?
